# Significantly smaller lateral extrusion was observed within 24 weeks after all‐inside suture repairs of radial tear in the middle segment of lateral meniscus compared to inside‐out repairs

**DOI:** 10.1002/jeo2.70041

**Published:** 2024-10-15

**Authors:** Ryohei Uchida, Shuji Horibe, Yoshinari Tanaka, Akira Tsujii, Yuta Tachibana, Kazutaka Kinugasa, Yoshiki Shiozaki

**Affiliations:** ^1^ Department of Orthopaedic Sports Medicine Kansai Rosai Hospital Amagasaki Japan; ^2^ Department of Orthopaedic Sports Medicine Seifu Hospital Sakai Japan; ^3^ Department of Nutrition, Graduate School of Human Life and Ecology, Osaka Metropolitan University Habikino Japan; ^4^ Department of Orthopaedic Surgery Osaka University Graduate School of Medicine Suita Japan; ^5^ Department of Orthopaedic Sports Medicine Osaka Rosai Hospital Sakai Japan

**Keywords:** all‐inside suture repair, inside‐out repair, lateral meniscus, meniscal extrusion, MRI

## Abstract

**Purpose:**

To evaluate the postoperative meniscal extrusion between all‐inside suture (AIS) and trans‐capsular suture (TCS) repair techniques.

**Methods:**

Thirteen patients (mean age, 19.4 years) underwent AIS repairs using only sutures (AIS group) for radial tears in the middle segment of the lateral meniscus (RTMLM), and seven patients (mean age, 20.3 years) underwent inside‐out repairs among TCS (TCS group). For all cases, lateral (LE), anterior (AE) and posterior (PE) meniscal extrusions of the lateral meniscus were measured during preoperative and 3‐, 12‐ and 24‐week postoperative MRIs. Then, the change of each extrusion from preoperative to each postoperative period was calculated as ∆LE, ∆AE and ∆PE.

**Results:**

There was no significant difference between the AIS and TCS groups in the preoperative extrusions. As for postoperative extrusions, only ∆LEs in the AIS group was significantly smaller than those in the TCS group at all postoperative periods (−1.5 ± 1.7 vs. 0.9 ± 0.7 mm at 3‐week, −0.9 ± 0.9 vs. 0.4 ± 0.9 mm at 12‐week and −0.3 ± 1.0 vs. 0.6 ± 1.1 mm at 24‐week postoperation). In ∆AEs and ∆PEs, at all three postoperative periods, there were no significant differences.

**Conclusion:**

Postoperative LE, AE and PE on MRIs after AIS and TCS repairs for RTMLM were investigated. Significantly smaller lateral extrusion was observed within 24 weeks after AIS repairs of RTMLM compared to TCS repairs, which could lead to stabilization of the repair site and prevent degenerative changes.

**Level of Evidence:**

Case‐control study, retrospective comparative study, Level Ⅲ.

**Abbreviations**
∆AE: delta anterior meniscal body extrusion
∆LE: delta lateral meniscal body extrusion
∆PE: delta posterior meniscal body extrusion
AE: anterior meniscal body extrusion
AIS: all‐inside suture
LE: lateral meniscal body extrusion
PE: posterior meniscal body extrusion
ROM: range‐of‐motion
RTMLM: radial tears in the middle segment of the lateral meniscus
TCS: trans‐capsular suture

## INTRODUCTION

Radial tears in the middle segment are the most common type of traumatic lateral meniscus injury in young athletes [15]. Conventionally, meniscectomy was the first‐line treatment for this type of tear, but it did not prevent degenerative changes or the decrease in meniscal function [6, 13]. Several studies have reported the use of meniscal repair of radial tears in the middle segment of the lateral meniscus (RTMLM) involving the vascular zone as an alternative treatment option [2, 4, 16, 19]. Two repair techniques have been described, including (1) the conventional trans‐capsular suture (TCS) repair technique, which includes inside‐out and outside‐in repair, and (2) the all‐inside suture (AIS) repair technique, which requires sutures but no TCS. Both techniques can achieve satisfactory clinical results, but complete healing rates on second‐look arthroscopy were low after RTMLM repairs [2, 4, 16, 19].

In our recent comparative study, AIS and TCS repairs for RTMLM were comparable in providing satisfactory clinical results [19]. However, the failure rate after AIS was significantly lower than that after TCS, suggesting some advantages of AIS over TCS for RTMLM repair. Supporting this, AIS repair minimized meniscal body extrusion more effectively at 24 weeks after repair than TCS repair in that study. In a biomechanical study, meniscal extrusion was reported to be related to tibiofemoral contact area and pressure [9]. Therefore, in clinical settings, investigating meniscal body extrusion is important for better understanding the extent of functional restoration of the repaired meniscus. We previously speculated that AIS repair could apply a direct compression force against the tear site, whereas after TCS repair, there could be traction of stumps to the periphery by the sutures passed through the capsule immediately after repair [19]. However, in that study, extrusion was evaluated only by 24‐week MRIs, and only ‘lateral’ meniscal body extrusion (LE) on the coronal plane was evaluated. For meniscus healing, it is important to clarify the amount and change of meniscal bony extrusion during the early postoperative period. To gain a deeper understanding of the effects of each suture technique, it will be meaningful to examine morphological changes that occur in the entire lateral meniscus. In other words, rather than limiting the evaluation to the LE on the coronal plane, anterior and posterior meniscal body extrusions (AE and PE) on the sagittal plane should also be evaluated by MRI.

To this end, this study aimed to evaluate and compare chronological changes that occur in lateral, anterior and posterior meniscal extrusion postoperatively between AIS and TCS repairs for RTMLM. We hypothesized that postoperative LE, AE and PE after the AIS repair technique would be smaller compared to preoperative LE, AE and PE, while those after the TCS repair technique would be larger, and that these changes would gradually diminish over time.

## MATERIALS AND METHODS

### Patients

This study was approved by the institutional review board of our hospital (approval ID: 20‐3). This was a retrospective study with a historical control group.

In this study, 47 patients after repair of isolated RTMLM were included from October 2011 to December 2019 at our institution. Inclusion criteria were as follows: (1) traumatic meniscus injury, (2) contralateral healthy knee, (3) no other meniscus and ligament injury, (4) no radiological osteoarthritic changes. Patients with tears involving only the white‐white zone, tears of the anterior or posterior horns, other tear types in the middle segment of the lateral meniscus, and medial meniscus tears were excluded. Twenty patients underwent preoperative and 3‐, 12‐ and 24‐week postoperative MRI evaluations. Seven patients (mean age, 20.3 years; range, 15–31 years) underwent TCS repair from October 2011 to July 2016 and 13 patients (mean age, 19.4 years; range, 18–22 years) underwent AIS repair from August 2016 to December 2019. Regardless of the repair techniques, all tears were full radial tears reaching the periphery in the red‐red zone and all the meniscus repairs were performed by two expert surgeons who each had more than 15 years of experience in arthroscopy. Patient demographic data are shown in Table 1.

**Table 1:** Patient demographic data.

	AIS group	TCS group	*p*‐Value
Number of patients	13	7	
Sex (male/female)	12: 1	6: 1	0.639
Age (years)	19.4 ± 2.0	20.3 ± 2.2	0.366
Body mass index (kg/m^2^)	26.3 ± 3.8	25.2 ± 3.5	0.534
Time from injury to repair (weeks)	3.7 ± 5.6	6.2 ± 1.0	0.262
Average operating time (minutes)	82 ± 37	76 ± 25	0.707
Number of average sutures	2.4 (2–3)	4.5 (4–5)	<0.001
Follow‐up time (months)	25.1 ± 2.8 (24–38)	25.4 ± 2.6 (24–32)	0.818

Abbreviations: AIS, all‐inside suture repair; TCS, trans‐capsular suture repair.

### Surgical procedure

In both groups, arthroscopic evaluation was performed using two standard anterior knee arthroscopy portals. After evaluating cruciate ligaments and chondral status, meniscal repair was performed with an additional far anteromedial portal in the figure‐four position.

**Figure 1:**
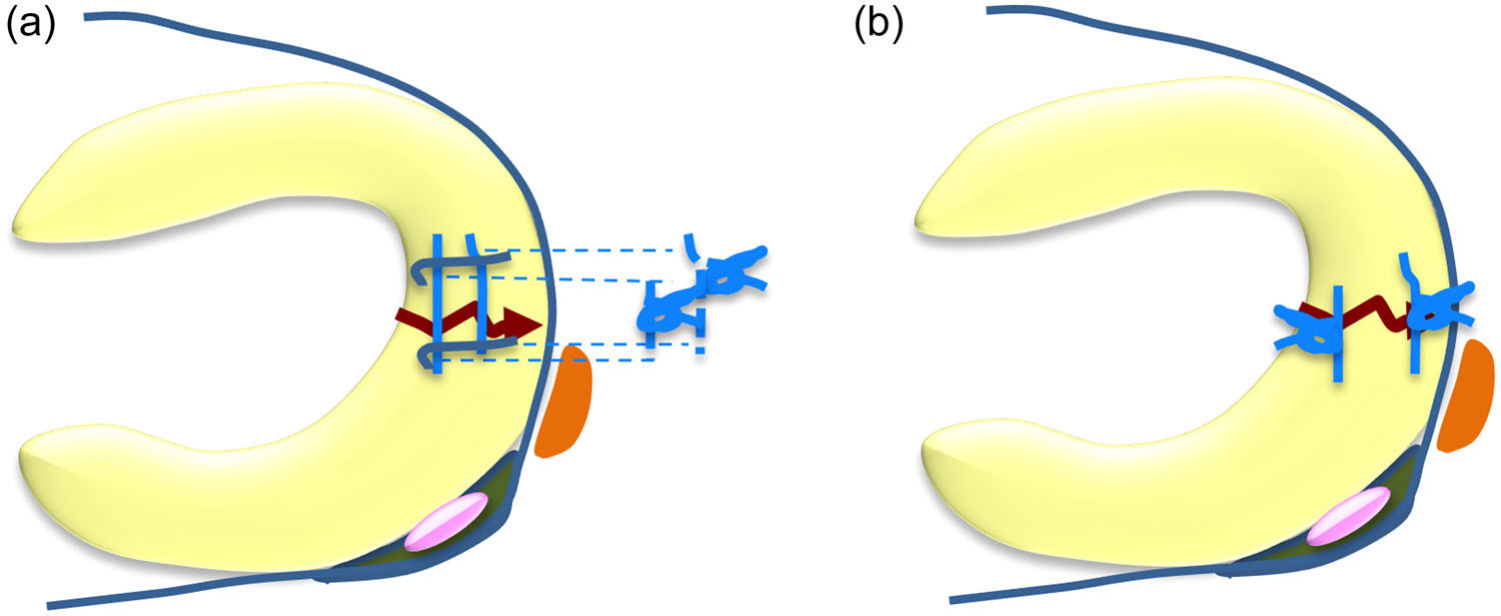
Meniscal repair technique. Schematic diagrams of tie‐grip sutures using the inside‐out repair technique (a) and double‐horizontal sutures using the all‐inside suture repair technique (b).

In the TCS group (Figure 1a), the inside‐out ‘tie‐grip suture’ technique was used with 2‐0 nonabsorbable Ethibond sutures (Johnson & Johnson) [12, 17]. A skin incision (3–4 cm in length) was also made at the lateral aspect of the knee to avoid neurovascular injury, and all sutures were tied against the joint capsule (trans‐capsular suturing). This technique involves the use of at least four sutures: two stay sutures placed vertically and at least two suture loops placed horizontally. Vertical sutures are necessary as grips to prevent cutting and slipping of the horizontal sutures that follow.

In the AIS group (Figure 1b) [18], the initial two penetration points by Suture hook or SutureLasso (Arthrex) for both the anterior and posterior stumps of the torn meniscus were carefully chosen and the device was inserted into the stumps. After suture relay, a 2‐0 nonabsorbable FiberWire suture (Arthrex) was inserted through the anterior and posterior stumps, and then knot‐tying was performed through a cannula. The number of sutures is determined according to the size of the tear.

### Postoperative regimen

The postoperative rehabilitation protocol was the same in both groups. The knee was immobilized with a brace for two weeks. Range‐of‐motion (ROM) exercises were started at 2 weeks and gradually increased, while the knee flexion angle was limited to 60° from 2 to 3 weeks postoperatively, 90° from 3 to 4 weeks postoperatively, and 120° from 4 to 24 weeks postoperatively. Partial and full weight‐bearing was permitted at 6 and 8 weeks postoperatively, respectively. Sports activities were allowed after second‐look arthroscopy.

### MRI evaluation

**Figure 2:**
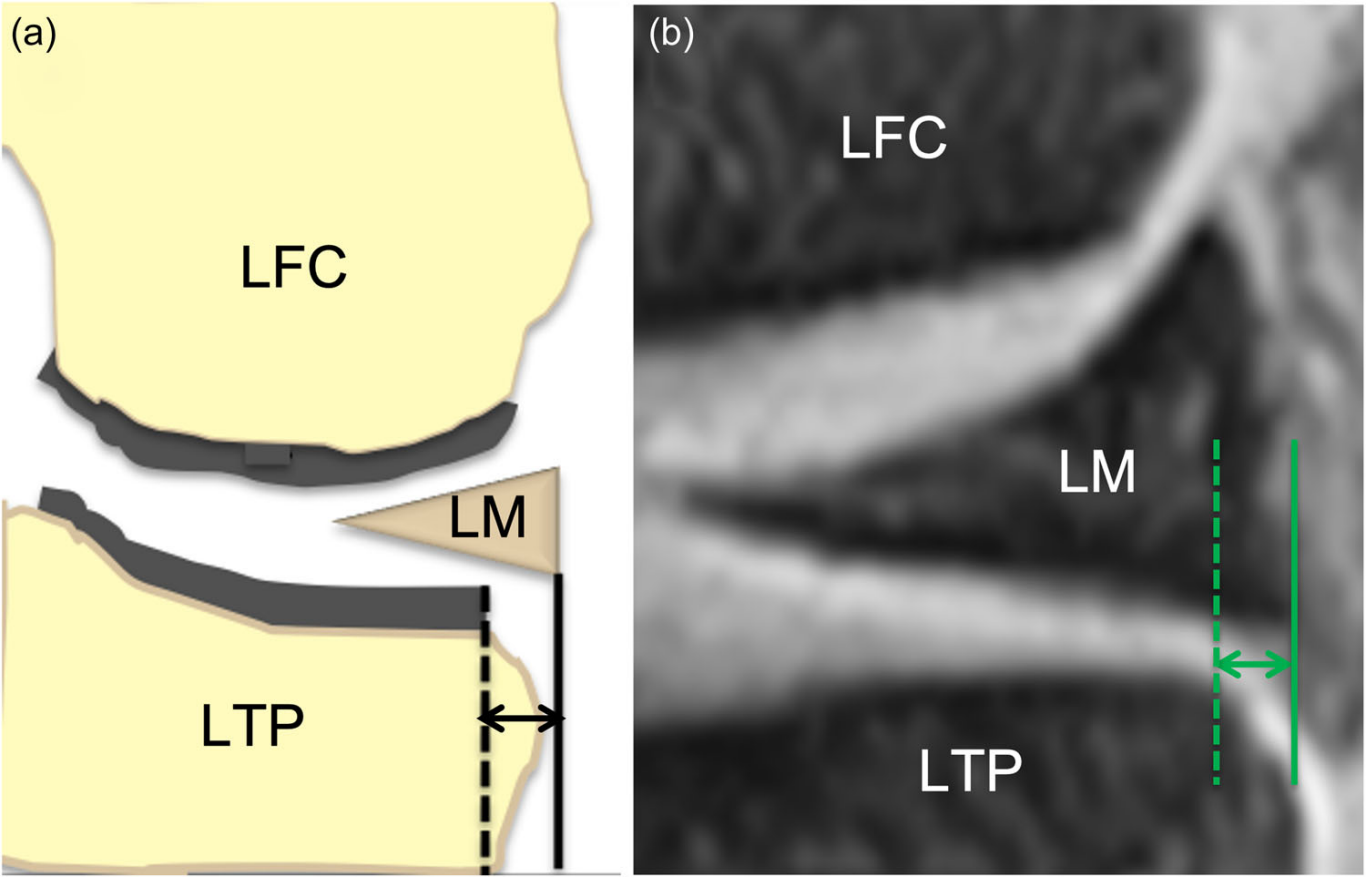
Measurement of lateral meniscal body extrusion. Schematic diagram (a) and MRI (b) for measurements of lateral meniscal body extrusion on the mid‐coronal plane. A black or green dotted line was perpendicular to the line from the medial to lateral osteochondral junctions through the outer edge of the lateral articular cartilage of the lateral tibial plateau. A solid black or green line was also perpendicular to the same line through the outer edge of the lateral meniscus. Then, the distance between solid line and dotted line (black or green arrow) was measured as a lateral meniscal body extrusion. LFC, lateral femoral condyle; LM, lateral meniscus; LTP, lateral tibial plateau.

**Figure 3:**
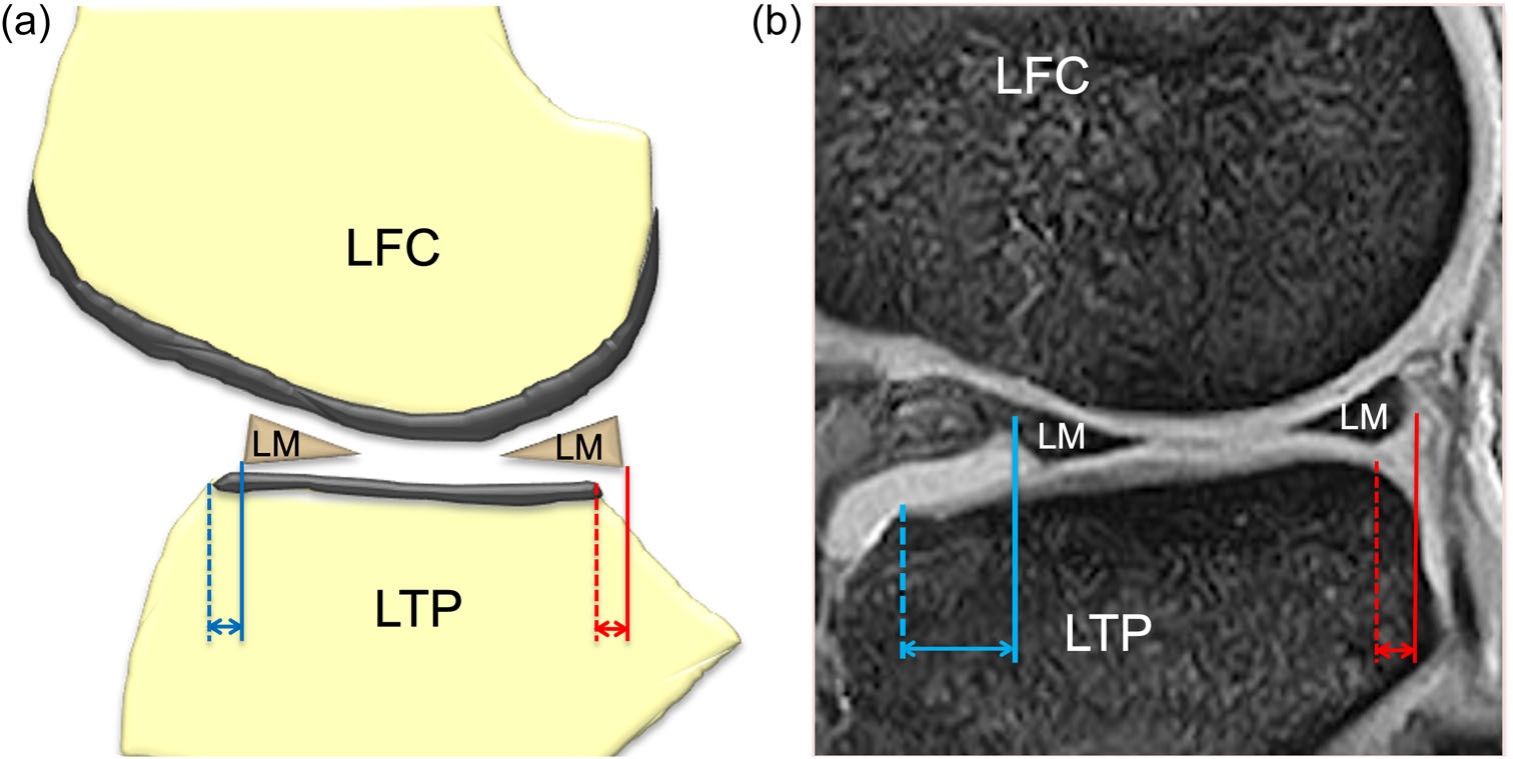
Measurement of anterior and posterior meniscal body extrusions. Schematic diagram (a) and MRI (b) for measurements of anterior and posterior meniscal body extrusions on the mid‐sagittal plane. A blue or red dotted line was perpendicular to the line from the osteochondral junctions through the anterior or posterior edge of articular cartilage of the lateral tibial plateau. A solid blue or red line was also perpendicular to the same line through the anterior or posterior edge of the lateral meniscus. Then, the distance between the solid line and dotted line (blue or red arrow) was measured as an anterior or posterior meniscal body extrusion. LFC, lateral femoral condyle; LM, lateral meniscus; LTP, lateral tibial plateau.

Preoperative and 3‐, 12‐ and 24‐week postoperative 1.5‐T MRI examinations were performed in all patients by the two orthopaedic surgeons. T2‐weighted images were obtained (repetition time, 500 ms; echo time, 14 ms) using a 16‐cm field of view and 512 × 512 matrix. Slices were 3.5 mm in thickness, with a gap of 0.5 mm. Before measurements of meniscal extrusion, the mid‐coronal plane and mid‐sagittal plane were determined according to a previous report [5]. Both mid‐coronal and sagittal planes through the center of the lateral femoro‐tibial compartment were identified by counting the total number of slices through the compartment and selecting the middle slice. Then, the distance between the outer edge of the articular cartilage of the tibial plateau and the outer edge of the lateral meniscus was measured as the LE on the mid‐coronal plane (Figure 2) [5, 7]. Distances from the anterior or posterior edge of the articular cartilage of the plateau to the anterior or posterior edge of the lateral meniscus were measured as the AE or PE on the mid‐sagittal plane, respectively (Figure 3) [5, 10]. The difference from pre‐ to each postoperative extrusion was calculated as delta extrusion (∆LE, ∆AE and ∆PE). OsiriX software (version 7.0.3; Pixmeo) was used to measure the meniscal extrusions. The reliability calculations were performed on the AE, LE and PE, and the intra‐ and interobserver intraclass correlation coefficient of AE, LE and PE were 0.88–0.95.

### Statistical analysis

Statistical analysis was performed using SPSS (SPSS Inc.). The Mann–Whitney *U* test was used to evaluate differences between the two groups, and the Friedman test was used to compare preoperative, 3‐, 12‐ and 24‐week postoperative values in each group. Pearson's correlation coefficients were used to assess the correlation among ∆LE, ∆AE and ∆PE. *p* < 0.05 was considered statistically significant. In the results of two‐group comparison on delta extrusions, post hoc power calculation was performed using a free power analysis program (G*Power 3.1).

## RESULTS

There were no significant differences between the two groups for all preoperative extrusions (Table 2). Postoperative changes in meniscal extrusion compared to preoperative meniscal extrusion are shown in Table 3. There were no significant correlations among ∆LE, ∆AE and ∆PE.

**Table 2:** Preoperative meniscal extrusion.

	AIS group	TCS group	*p*‐Value
Number of patients	13	7	
Lateral extrusion (mm)	2.7 ± 1.2	2.1 ± 0.7	0.292
Anterior extrusion (mm)	−2.5 ± 1.9	−4.3 ± 1.8	0.191
Posterior extrusion (mm)	−0.4 ± 1.6	−0.7 ± 1.9	0.382

Abbreviations: AIS, all‐inside suture repair; TCS, trans‐capsular suture repair.

**Table 3:** Postoperative change of meniscal extrusion.

		AIS group	TCS group	*p*‐Value
Number of patients		13	7	
ΔLE (mm)	3 wks.	−1.5 ± 1.7^a^	0.9 ± 0.7^a^	0.001
	12 wks.	−0.9 ± 0.9^a^, ^b^	0.4 ± 0.9^a^, ^b^	0.001
	24 wks.	−0.3 ± 1.0^b^	0.6 ± 1.1^b^	0.04
ΔAE (mm)	3 wks.	−0.2 ± 0.9	0.5 ± 0.7	0.29
	12 wks.	0.6 ± 1.4^b^	1.3 ± 1.2^a^	0.22
	24 wks.	1.0 ± 1.2^a^, ^b^	2.5 ± 1.6^a^, ^b^, ^c^	0.07
ΔPE (mm)	3 wks.	2.0 ± 1.9^a^	−1.2 ± 1.0^a^	0.73
	12 wks.	−0.6 ± 0.9^a^	−0.4 ± 1.0^b^	0.97
	24 wks.	−0.3 ± 1.3^b^	0.4 ± 1.2^b^	0.83

Abbreviations: AIS, all‐inside suture repair; TCS, trans‐capsular suture repair; wks, weeks; ΔAE, change of anterior extrusion from preoperative to each postoperative period; ΔLE, change of lateral extrusion from preoperative to each postoperative period; ΔPE, change of posterior extrusion from preoperative to each postoperative period.

^a^ Significant difference between preoperative and each postoperative meniscus extrusion.

^b^ Significant difference 3‐week postoperative and 12‐ or 24‐week postoperative meniscus extrusion.

^c^ Significant difference 12‐week postoperative and 24‐week postoperative meniscus extrusion.

### Postoperative change of LE

As for the change of ∆LE, ∆LE in the TCS group increased to the peak value at 3 weeks postoperatively, while ∆LE in the AIS group decreased to the peak value at 3 weeks postoperatively. From 3 to 24 weeks postoperatively, ∆LEs in both groups were near the preoperative state. Comparing the two groups, ∆LE in the AIS group was significantly smaller than that in the TCS group at each postoperative period (*p* = 0.001 at 3 weeks, *p* = 0.001 at 12 weeks, and *p* = 0.04 at 24 weeks). After post hoc power calculation, on 3‐ and 12‐week postoperative MRI evaluations, sufficient statistical powers were 0.99 and 0.81, respectively, although a statistical power was only 0.39 on 24‐week MRI evaluations.

**Figure 4:**
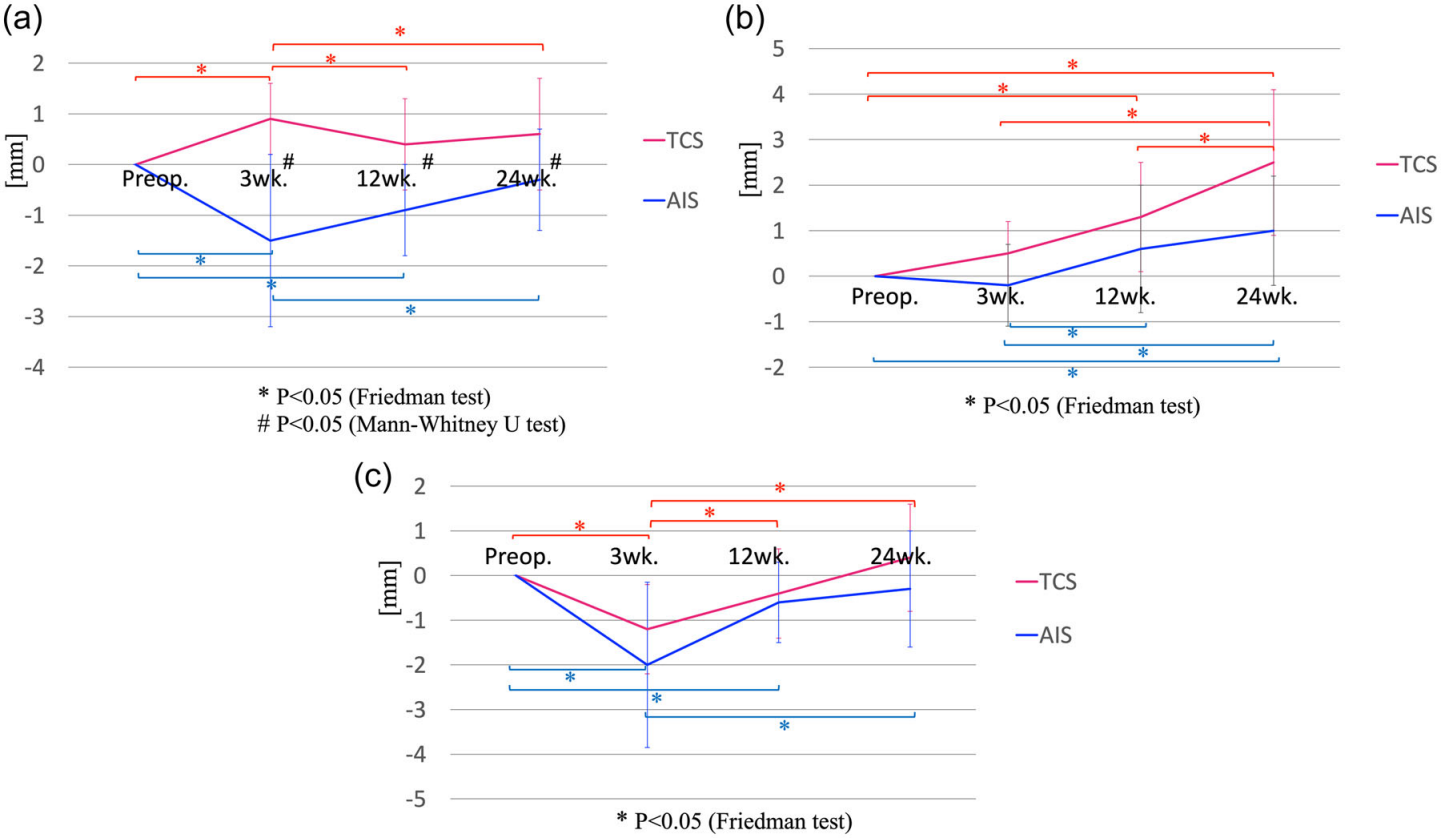
Postoperative change of meniscal extrusions. (a) Postoperative change of lateral meniscal extrusion (∆LE). (b) Postoperative change of anterior meniscal extrusion (∆AE). (c) Postoperative change of posterior meniscal extrusion (∆PE). AIS, all‐inside suture repair; pre‐op., preoperation; TCS, trans‐capsular suture repair; wk, week. *Significant difference between preoperative and each postoperative meniscus extrusion (*p* < 0.05). ^**#**^Significant difference in postoperative meniscus extrusion between AIS and TCS at each postoperative period (*p* < 0.05).

Compared to the preoperative state, 3‐week postoperative ∆LE in the TCS group was significantly larger (*p* = 0.007), while 3‐ and 12‐week postoperative ∆LEs in the AIS group were significantly smaller (*p* < 0.001 at 3 weeks, *p* = 0.007 at 12 weeks). Moreover, compared to the 3‐week postoperative state, 12‐ and 24‐week postoperative ∆LEs in the TCS group were significantly smaller (*p* = 0.045 at 12 weeks, *p* = 0.001 at 24 weeks), while 24‐week postoperative ∆LE in the AIS group was significantly larger (*p* = 0.015) (Figure 4a).

### Postoperative change of AE

The change of ∆AEs in both groups showed a similar pattern. Specifically, ∆AEs in both groups gradually increased from preoperation to 24 weeks postoperatively. There were no significant differences in ∆AEs between the two groups at any assessed postoperative period. Compared to the preoperative state, 12‐ and 24‐week postoperative ∆AEs in the TCS group were significantly larger (*p* = 0.011 at 12 weeks, *p* < 0.001 at 24 weeks), while only 24‐week postoperative ∆AE in the AIS group was significantly larger (*p* = 0.017). Moreover, compared to the 3‐week postoperative state, 24‐week postoperative ∆AE in the TCS group was significantly larger (*p* = 0.001), while 12‐ and 24‐week postoperative ∆AEs in the AIS group were significantly larger (*p* = 0.018 at 12 weeks, *p* = 0.004 at 24 weeks). In TCS group, 24‐week ∆AE was significantly larger than 12‐week postoperative ∆AE (*p* = 0.024) (Figure 4b).

### Postoperative change of PE

The change of ∆PEs in both groups also showed a similar pattern. ∆PEs in both groups were negative values at 3 weeks postoperatively, but gradually increased with time through 24 weeks postoperatively. There were no significant differences in ∆PEs between the two groups at any assessed postoperative period. Compared to the preoperative state, 3‐week postoperative ∆PEs in the both groups were significantly different (*p* = 0.007 in TCS group, *p* < 0.001 in AIS group). Moreover, 12‐ and 24‐week postoperative ∆PEs in both groups were also significantly different compared to the 3‐week postoperative state (*p* = 0.045 at 12 weeks, *p* = 0.001 at 24 weeks in TCS group and *p* = 0.005 at 12 weeks, *p* < 0.001 at 24 weeks in AIS group) (Figure 4c).

## DISCUSSION

The main finding of this study was the difference in ΔLE between the TCS and AIS groups. From preoperation to 3 weeks postoperatively, LE in AIS group decreased, whereas it increased in the TCS group. In all assessed postoperative periods, ΔLE in the AIS group was significantly smaller than that in the TCS group. With regard to ΔAE and ΔPE, there were no significant differences between the two groups, although averages in the AIS group tended to be smaller than those in the TCS group.

According to a previous study, changes in lateral and posterior extrusions were weakly correlated with meniscus healing after longitudinal tear repair [17]. Although that study did not provide data on the relationship between meniscus healing and extrusion after radial tear repair, a smaller lateral extrusion after AIS repair would affect meniscus healing. Indeed, in our previous report, we observed a lower failure rate after AIS repair of RTMLM compared with that after TCS repair [19].

**Figure 5:**
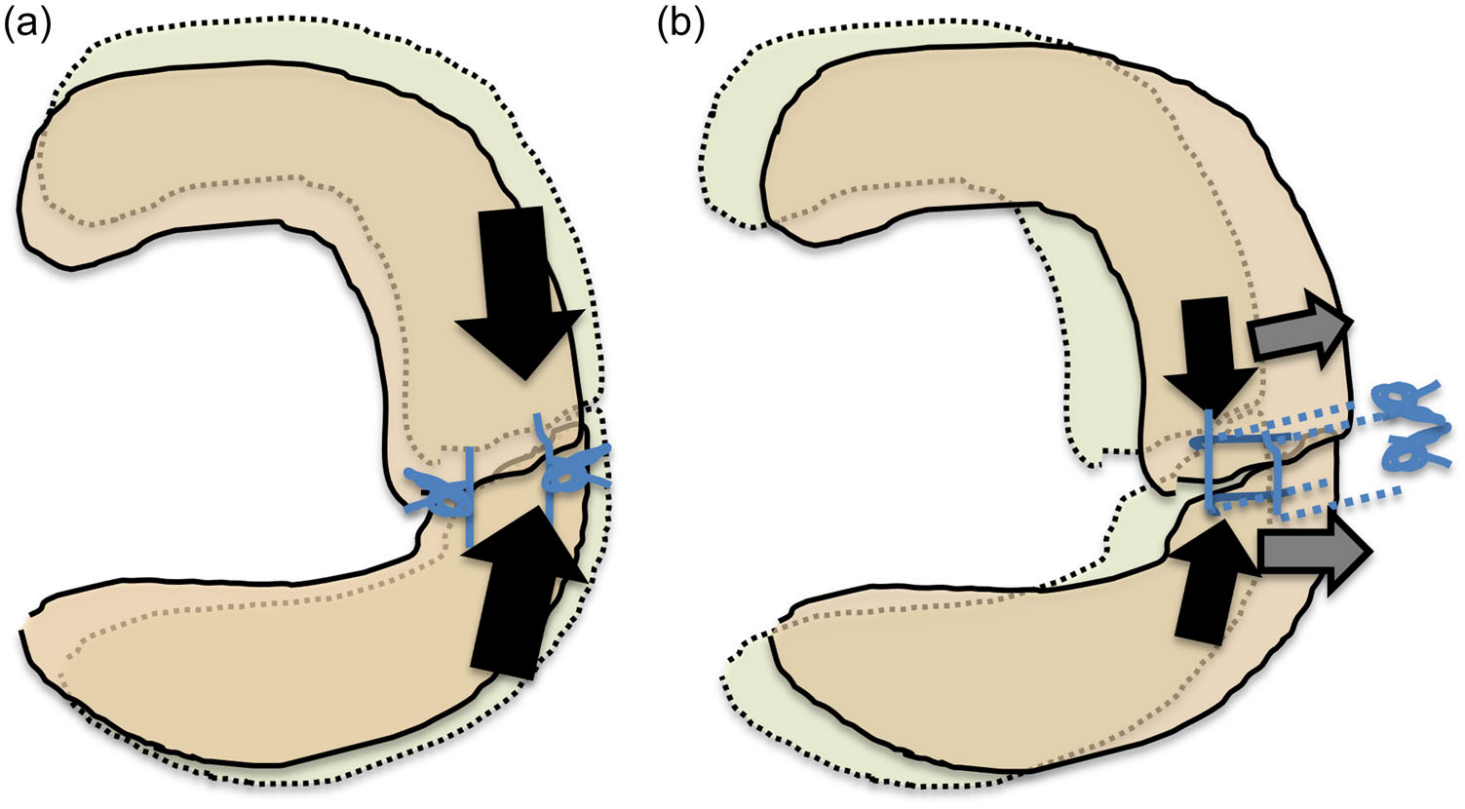
Effects of repair techniques on morphological change of lateral meniscus in early postoperative period. (a) From preoperation to 3‐week postoperation after AIS repair, ∆LE and ∆PE were significantly decreased in all cases. In AIS repair, an intrameniscus suturing without penetrating the capsule could apply only direct compressive force (black arrows) against the torn edges to sustain the stability of the repair site [19]. As a result, the whole lateral meniscus body would be restored like a normal uninjured meniscus. Therefore, both extrusions of the middle segment and the posterior segment were diminished at 3‐week postoperation. (b) From preoperation to 3‐week postoperation after TCS (inside‐out) repair, ∆LE was significantly increased and ∆PE was decreased. In TCS repair, there could be not only direct force (black arrows) against the torn edges but also tensile force (grey arrows) to the periphery by the sutures penetrating the capsule [19]. This tensile force in TCS repair may lead the whole lateral meniscus body towards the lateral outer direction. As a result, extrusion of the middle segment was increased and extrusion at the posterior segment was conversely diminished. AIS, all‐inside suture repair; TCS, trans‐capsular suture repair.

Focused on the change of postoperative meniscus extrusions, we divided two periods as follows in consideration with the change of ∆LE (1) preoperation to 3 weeks postoperation and (2) 3 to 24 weeks postoperation. From preoperation to 3 weeks postoperation, ∆LE and ∆PE were significantly decreased in the AIS group, whereas ∆LE was significantly increased in the TCS group. In our previous study [19], the resulting reactive force of the stitch of AIS is made directly opposite to the direction of displacement, whereas an inside‐out repair stitch is made oblique to it. Intrameniscus suturing without a penetrating capsule in AIS applies only direct compressive force against the torn edges to sustain the stability of the repair site. As a result, meniscal body extrusion due to meniscus tear was decreased at 3 weeks postoperation (Figure 5a). In contrast, after TCS repair, there was not only direct force against the torn edges but also tensile force to the periphery by the suture penetrating capsule. This tensile force to the periphery in TCS repair may have led to the loss of stability at repair and the increase in 3‐week postoperative ∆LE (Figure 5b). Previous cadaveric and experimental studies revealed that AIS repair of radial meniscus tears had biomechanical superiority with a smaller gap after cyclic loading as well as a larger failure load and less stiffness compared with TCS repair [1, 3, 8]. These biomechanical features of suture techniques could contribute to the ∆LE we observed in this study. In particular, at 3 weeks postoperation, ROM exercises had already started. For the meniscus, ROM exercises may be similar to being under cyclic loading.

After 3 weeks postoperation, focused on the period from 3‐ to 12‐week postoperation, ∆LEs, ∆PEs and ∆AEs in both groups, except ∆AE in the TCS group, transitioned to the opposite direction compared to those from preoperation to 3 weeks postoperation. The postoperative load in the meniscal body would gradually relax due to the meniscal suturing, leading to changes in extrusion after 3 weeks postoperatively. From 3 to 24 weeks postoperation, ∆LEs, ∆PEs and ∆AEs in both groups changed significantly. Especially, ∆LEs, ∆PEs and ∆AEs in both group except ∆LE in the TCS group were significantly increased. For postoperative rehabilitation, >90° flexion and weight‐bearing were started after 3 weeks. An in vivo study using MRI showed the lateral meniscus to be laterally and posteriorly extruded under flexion and weight‐bearing conditions of the knee [11]. Although there was little evidence for how long it took for the meniscus to heal and how strong the healed tissue was, a previous study demonstrated that, even after 12 weeks, a repaired meniscus was still significantly weaker than a healthy meniscus [14]. After 3 weeks postoperation, the repaired meniscus with biomechanical weakness was always exposed to the force for meniscal extrusion. This may have led to the increase of ∆LE, ∆PE and ∆AE.

The present study has some limitations. First, since different surgical methods were used (double horizontal suture for AIS and tie‐grip suture for TCS), the method should be unified in both groups to clarify differences in meniscal extrusion between the two repair techniques. Second, the follow‐up period was only 24 weeks. A longer follow‐up period is necessary to determine whether meniscus repair can prevent postoperative degenerative changes for longer periods. Third, in this study, the meniscal extrusion was not compared between repaired meniscus and normal uninjured meniscus. Therefore, in the near future, the evaluation of meniscal extrusion in the healthy knee should be performed. Finally, as the sample size was small, more cases will need to be evaluated. But a total of four MRIs (preoperative and three postoperative MRIs) were taken per case in this study. The data from these cases are valuable, even though some of the results with significant difference had insufficient power. Moreover, the present study is the first to provide a chronological evaluation of meniscal extrusion after RTMLM repair and our data contribute to the current understanding of isolated RTMLM management.

## CONCLUSIONS

Postoperative LE, AE and PE on MRIs after AIS and TCS repairs for RTMLM were investigated. Significantly smaller lateral extrusion was observed within 24 weeks after AIS repairs of RTMLM compared to TCS repairs, which could lead to stabilization of the repair site and prevent degenerative changes.

## AUTHOR CONTRIBUTIONS

*Contributed to the conception and design*: Ryohei Uchida. *Carried out the patient's operation and acquisition of data*: Ryohei Uchida, Shuji Horibe, Yuta Tachibana, Kazutaka Kinugasa and Yoshinari Tanaka. *Responsibility for acquisition of data*: Ryohei Uchida and Yoshiki Shiozaki. *Analysis and critical interpretation of data, including review and evaluation of previous studies*: Shuji Horibe and Akira Tsujii. *Drafting the manuscript*: Ryohei Uchida. All authors read and approved the final manuscript.

## CONFLICT OF INTEREST STATEMENT

The authors declare no conflict of interest.

## ETHICS STATEMENT

Ethical approval for the study was obtained from the ethics committees of Seifu Hospital (ID: 20‐3).

## Data Availability

The data that support the findings of this study are available on request from the corresponding author. The data are not publicly available due to privacy or ethical restrictions.
